# Organization of sensorimotor activity in anterior cruciate ligament reconstructed individuals: an fMRI conjunction analysis

**DOI:** 10.3389/fnhum.2023.1263292

**Published:** 2023-11-24

**Authors:** Amber J. Schnittjer, HoWon Kim, Adam S. Lepley, James A. Onate, Cody R. Criss, Janet E. Simon, Dustin R. Grooms

**Affiliations:** ^1^Translational Biomedical Sciences, Graduate College, Ohio University, Athens, OH, United States; ^2^Ohio Musculoskeletal and Neurological Institute (OMNI), Ohio University, Athens, OH, United States; ^3^School of Kinesiology, Exercise and Sports Science Initiative, University of Michigan, Ann Arbor, MI, United States; ^4^School of Health and Rehabilitation Sciences, The Ohio State University, Columbus, OH, United States; ^5^OhioHealth Riverside Methodist Hospital, Columbus, OH, United States; ^6^Division of Athletic Training, School of Applied Health Sciences and Wellness, College of Health Sciences and Professions, Ohio University, Athens, OH, United States; ^7^Division of Physical Therapy, School of Rehabilitation and Communication Sciences, College of Health Sciences and Professions, Ohio University, Athens, OH, United States

**Keywords:** brain, knee, functional magnetic resonance imaging, motor control, rehabilitation

## Abstract

**Introduction:**

Anterior cruciate ligament reconstruction (ACLR) is characterized by persistent involved limb functional deficits that persist for years despite rehabilitation. Previous research provides evidence of both peripheral and central nervous system adaptations following ACLR. However, no study has compared functional organization of the brain for involved limb motor control relative to the uninvolved limb and healthy controls. The purpose of this study was to examine sensorimotor cortex and cerebellar functional activity overlap and non-overlap during a knee motor control task between groups (ACLR and control), and to determine cortical organization of involved and uninvolved limb movement between groups.

**Methods:**

Eighteen participants with left knee ACLR and 18 control participants performed a knee flexion/extension motor control task during functional magnetic resonance imaging (fMRI). A conjunction analysis was conducted to determine the degree of overlap in brain activity for involved and uninvolved limb knee motor control between groups.

**Results:**

The ACLR group had a statistically higher mean percent signal change in the sensorimotor cortex for the involved > uninvolved contrast compared to the control group. Brain activity between groups statistically overlapped in sensorimotor regions of the cortex and cerebellum for both group contrasts: involved > uninvolved and uninvolved > involved. Relative to the control group, the ACLR group uniquely activated superior parietal regions (precuneus, lateral occipital cortex) for involved limb motor control. Additionally, for involved limb motor control, the ACLR group displayed a medial and superior shift in peak voxel location in frontal regions; for parietal regions, the ACLR group had a more posterior and superior peak voxel location relative to the control group.

**Conclusion:**

ACLR may result in unique activation of the sensorimotor cortex via a cortically driven sensory integration strategy to maintain involved limb motor control. The ACLR group's unique brain activity was independent of strength, self-reported knee function, and time from surgery.

## 1 Introduction

Anterior cruciate ligament (ACL) rupture is a prevalent injury among physically active individuals, particularly those involved in running, cutting, and jumping sports (Boden et al., 2000; Besier et al., 2001). Currently, in the United States, the standard treatment after injury is anterior cruciate ligament reconstruction (ACLR), which restores mechanical stability to the knee joint. Approximately 200,000 ACL reconstructions are performed in the United States annually, resulting in a cost of $7.6 billion per year ($38,000/per surgery) (Mather et al., 2013). Unfortunately, individuals who undergo ACLR are at a 30–40 times greater risk of secondary ACL injury relative to healthy, uninjured individuals (Wiggins et al., 2016). Notably, this increased risk of secondary injury affects both the injured and uninjured limbs, with a recent meta-analysis reporting the ipsilateral ACL injury rate as 7% and the contralateral ACL injury rate as 8% (Wiggins et al., 2016). The heightened injury risk for both knees can be attributed to, at least in part, persistent central sensorimotor control deficits that remain months to years after reconstruction and rehabilitation (Baumeister et al., 2011; Grooms et al., 2017; An et al., 2019; Criss et al., 2020).

Indirect evidence suggests central nervous system (CNS) alterations associated with ACL injury may partially account for prolonged involved limb deficits (Kapreli et al., 2009; Grooms et al., 2017; Criss et al., 2022). Neuroimaging studies employing functional magnetic resonance imaging (fMRI) have examined both ACL-deficient (ACLD) and ACLR patients and revealed greater cortical activity in motor planning, visual processing, and multi-sensory integration regions of the brain during a motor task of the involved limb relative to controls (Kapreli et al., 2009; Grooms et al., 2017). These neural differences may contribute to prolonged deficits in quadriceps strength, functional performance, and joint loading after ACLR (Palmieri-Smith and Lepley, 2015; Schmitt et al., 2015; Lisee et al., 2022). A recent meta-analysis revealed quadriceps' strength and activation deficits in the involved limb of individuals with ACLR compared to their uninvolved limb and healthy controls persist years after surgery (Lisee et al., 2019). Furthermore, evidence indicates no direct association between quadriceps strength symmetry and the resolution of asymmetric gait mechanics in individuals with ACLR (Arhos et al., 2021). Although involved limb deficits are widely researched following ACLR, the underlying mechanism of these persistent asymmetries is not well understood.

While cortical activation differences have primarily been documented between the injured knee and healthy controls, patients with ACLR have also demonstrated altered spinal reflexive and corticospinal excitability between limbs (Kuenze et al., 2015; Zarzycki et al., 2020). For instance, Konishi et al. found gamma loop dysfunction of the quadriceps in both the involved and uninvolved limbs of patients with ACL rupture and ACLR (Konishi et al., 2003; Konishi, 2011). This between-limb comparison of Konishi et al.'s study highlights the need to consider comparisons of both the unaffected limb and healthy controls, which is a limitation of previous neuroimaging studies investigating cortical changes following ACLR and ACLD (Kapreli et al., 2009; Grooms et al., 2017). The spinal and cortical excitability and gamma motor data suggest both limbs are affected by neurological changes; however, involved limb sensorimotor cortical organization relative to the uninvolved limb and healthy controls is unknown. Gaining insight into the sensorimotor cortical organization associated with inter-limb motor control after ACL injury and reconstruction could provide valuable data to inform strategies to resolve treatment-resistant functional asymmetries. Hence, the objective of this study was to identify brain regions of functional activity overlap and non-overlap during an involved [left] and uninvolved [right] knee motor control task. Second, we aimed to explore cortical organization in patients with ACLR relative to healthy controls for involved and uninvolved knee movement. To capture potential driving factors of organizational differences between groups, behavioral outcome measures of self-reported knee function, pain, and quadriceps strength were also assessed. Based on prior investigations, we hypothesized that the ACLR group would have higher brain activity for involved limb movement and would activate more multi-sensory integration regions relative to the control group.

## 2 Materials and methods

### 2.1 Participants

Participants were recruited from the local university and nearby orthopedic clinics and were ultimately enrolled at two different neuroimaging sites (Ohio State University Center for Cognitive and Behavior Brain Imaging and the University of Connecticut Brain Imaging Research Center). As this was a secondary analysis from a larger study, an *a priori* power analysis was not conducted. Similar inclusion and exclusion criteria were used in previous neuroimaging studies investigating patients with ACLR (Grooms et al., 2017; Criss et al., 2022). Specifically, participants were included if they had a history of left primary unilateral ACLR and were between the ages of 16 and 35. Control participants were excluded if they had a history of previous orthopedic surgery or lower extremity injury within 6 months, a current diagnosis or history of concussion within 6 months, a history of stroke, migraines, or a neurological or psychiatric disorder. Participants were also excluded if they were experiencing any acute pain (i.e., headache and back pain). Participants in the ACLR group were 6 months to 5 years post-surgery, completed rehabilitation, and were cleared for full activity by their surgeons. Participant activity level was determined using the Tegner activity level scale (Tegner and Lysholm, 1985). Limb dominance was assessed for each participant as the preferred leg to kick a ball. The same number of right- and left-limb dominant individuals were recruited for each group. All participants received written and informed consent, and all procedures were approved by the Institutional Review Boards of both universities.

### 2.2 Behavioral outcome measures

#### 2.2.1 Patient-reported outcome measures

Self-reported knee function was assessed for each participant using two reliable and validated clinical scales: the International Knee Documentation Committee (IKDC) Subjective Form and the Knee Injury and Osteoarthritis Outcome Score (KOOS) (Roos et al., 1998; Irrgang et al., 2001; Higgins et al., 2007; Salavati et al., 2011). The IKDC provides information regarding overall knee function (Irrgang et al., 2001), while the KOOS provides subjective values for levels of pain, disease-specific symptoms, activities of daily living, sport and recreation function, and knee-related quality of life (Roos et al., 1998). For the purposes of this study, the KOOS Pain subscale was chosen as a primary variable of interest as we were interested in the effects of potential persistent or chronic pain on brain activity and cortical organization as it relates to ACLR. Prior studies examining other orthopedic conditions have well-established the link between chronic pain and cortical reorganization (Tsao et al., 2008, 2011; Shanahan et al., 2015; Te et al., 2017). It is important to note that neither the ACLR patients nor the control subjects experienced any pain during the neuroimaging paradigm described below.

#### 2.2.2 Quadriceps strength

Quadriceps muscle strength for both ACLR and control groups was measured bilaterally using isokinetic maximal voluntary contractions (MVCs). Participants were secured using both shoulder and lap straps (Biodex Medical Systems 4, Shirley, New York, USA) with the hips and testing knee secured at 90° of flexion. During testing, participants were instructed to cross their arms over their chests and extend their legs to ~0° knee extension. All participants completed a standardized warm-up of three submaximal contractions, followed by three isokinetic MVC trials (60°/s) with visual and verbal feedback to encourage maximal effort. Maximal torque was averaged over the three testing trials and normalized to body mass (Nm/kg) for analysis.

### 2.3 Neuroimaging paradigm

Functional magnetic resonance imaging (fMRI) data were collected from two separate neuroimaging centers following comparable methodology and scan parameters used in previous fMRI research (Criss et al., 2022). Each participant was placed supine in the scanner with their legs placed on a cushioned bolster that limited knee flexion to 45°. Participants' heads were secured and sufficiently padded to reduce head motion during the movement task. Splints were applied to the ankles and feet to limit accessory motion, and straps were placed over the thighs, pelvis, and torso to reduce accessory movement. Participants then completed a series of unilateral knee flexion/extension movements (knee motor control task). For the purposes of this study, the involved limb for the ACLR group was synonymous with the left limb for the control group, as all participants in the ACLR group had a surgical left knee. The uninvolved limb for the ACLR group was the same as the right leg for the control group. Limb dominance between groups was equal, with 16 right-limb dominant individuals and two left-limb dominant individuals per group. The movement frequency for the motor control task was controlled via an auditory metronome set at 1.2 Hz. Movement and rest blocks consisted of 30-s blocks (four blocks of knee flexion/extension movement and five blocks of rest). Previous fMRI studies have validated this movement paradigm in its ability to assess brain activation for knee movement and limit participant head motion (Kapreli et al., 2006, 2007; Grooms et al., 2017). Participants also received a full practice session in a mock scanner prior to data collection for familiarization with the task and were provided verbal instruction to limit head movement. All participants completed the task bilaterally, and each subject's data were averaged across trials.

### 2.4 Statistical analysis

Descriptive statistics were calculated for demographics and behavioral outcomes measures of strength, IKDC, and KOOS Pain using SPSS version 27 (SPSS Inc., Chicago, IL). Demographics and behavioral measures were assessed for the assumptions of normality and homogeneity of variances. Age, Tegner, IKDC, and KOOS Pain violated the assumption of normality and non-parametric Mann–Whitney *U*-tests were conducted (*p* < 0.05). Independent *t*-tests were used to determine whether there were statistically significant mean differences between ACLR and control groups for height, weight, involved limb MVC, and uninvolved limb MVC (*p* < 0.05). Data were presented as either mean ± standard deviation for parametric tests or median and interquartile range (IQR) for non-parametric tests.

### 2.5 fMRI statistical analysis

#### 2.5.1 First-level analysis

Neuroimaging data were preprocessed using the software package Oxford Center for Functional MRI of the Brain Software Library (FSL) 6.0 (FMRIB, Oxford, UK). The preprocessing pipeline included brain extraction, motion correction, 6-mm spatial smoothing, mean-based intensity normalization of all volumes, denoising with an independent component analysis-based strategy for automatic removal of motion artifacts (ICA-AROMA), high-pass temporal filtering at 100 s, and linear anatomical and non-linear standard space registration (Woolrich et al., 2001; Jenkinson et al., 2002; Smith, 2002; Pruim et al., 2015a,b).

A first-level analysis was completed for all participants to contrast between two conditions: condition 1 (rest) and condition 2 [task (knee motor control)]. This was done separately for both the involved [left] and uninvolved [right] limbs to measure average brain activity during knee motor control for each limb. All fMRI data analyses were conducted with an *a priori* threshold of *z* > 3.1, *p* < 0.05, and random field cluster correction, which are standard imaging parameter thresholds used to correct for multiple comparisons (Woolrich et al., 2001, 2004a,b; Smith et al., 2004; Eklund et al., 2016).

#### 2.5.2 Second-level analysis

To lateralize brain activity of the sensorimotor cortex and cerebellum to determine functional organization, a second-level analysis was conducted to compare brain activity between limbs during knee motor control using two contrasts: contrast 1 (involved [left] > uninvolved [right]) and contrast 2 (uninvolved [right] > involved [left]). In simple terms, these contrasts examine areas of greater activation when the involved limb is moving relative to the uninvolved limb and vice versa. This process was done separately for the ACLR and control groups. A mixed-effects FLAME1 + 2 model was used to determine second-level blood oxygen level-dependent (BOLD) activation.

The mean percent signal change, or change in BOLD signal between rest and movement, was extracted from each participant using FSL's *featquery* command to compare the intensity of brain activity between groups. This was done for the resulting brain activity from both contrasts in the second-level analysis (involved [left] > uninvolved [right]) and (uninvolved [right] > involved [left]). In total, four variables were analyzed and compared between ACLR and control groups: sensorimotor strip (involved [left] > uninvolved [right]), cerebellum (involved [left] > uninvolved [right]), sensorimotor strip (uninvolved [right] > involved [left]), and cerebellum (uninvolved [right] > involved [left]). Assumptions of normality and homogeneity of variances were evaluated for each dependent variable. The mean percent signal change for the cerebellum (uninvolved [right] > involved [left]) violated normality, and thus, a non-parametric Mann–Whitney *U*-test was conducted for that variable. The remaining three variables were compared using independent *t*-tests. *p*-values were corrected using the Benjamini–Hochberg procedure with a false discovery rate for multiple comparisons within each contrast (Benjamini and Hochberg, 1995). Corrected *p*-values were considered significant if they were < 0.05. For the purposes of this study, the sensorimotor strip was defined as the primary motor and sensory cortical regions.

#### 2.5.3 Conjunction analysis

A conjunction analysis was conducted to determine the degree of statistical overlap in brain activity between ACLR and control groups (*z* > 3.1, *p* < 0.05 cluster corrected). This was carried out for both second-level contrasts: (involved [left] > uninvolved [right]) and (uninvolved [right] > involved [left]). To determine unique non-overlapping areas of brain activity between ACLR and control groups, the *fslmaths* command was used to create unique masks by subtracting the overlapping clusters from each group's second-level analysis of brain activity. The unique non-overlapping areas of the sensorimotor strip were then split into frontal and parietal regions to more accurately isolate and define anatomical regions. The unique non-overlapping areas of the cerebellum were not split. FSL's *atlasquery* command was used to determine the anatomical regions within the overlapping and unique non-overlapping regions for both groups. Anatomical locations were identified using probabilistic maps derived from the Harvard-Oxford, Juelich, and Cerebellar Atlas in MNI 152 (FNIRT) atlases (Eickhoff et al., 2005, 2006, 2007; Frazier et al., 2005; Desikan et al., 2006; Makris et al., 2006; Goldstein et al., 2007; Diedrichsen et al., 2009). A probabilistic threshold of ≥5% was used to determine anatomical areas included in the sensorimotor strip clusters, and ≥1% was used to determine anatomical areas in the cerebellar clusters.

To determine sensorimotor strip organization between groups, FSL's *featquery* was used to obtain each subject's peak MNI voxel coordinates for the frontal and parietal non-overlapping clusters. The MNI peak voxel coordinates were interpreted as *x* (medial–lateral), *y* (anterior–posterior), and *z* (superior–inferior). There were 12 dependent variables [peak voxel location (*x, y, z*) for the non-overlapping areas in the sensorimotor strip (frontal and parietal)] for each second-level contrast (involved [left] > uninvolved [right] and uninvolved [right] > involved [left]). Descriptive statistics were calculated for each dependent variable by group using SPSS. Assumptions of normality and homogeneity of variances were evaluated for each dependent variable. Six dependent variables violated normality for at least one of the groups (involved [left] > uninvolved [right]: frontal *y*, frontal *z*, and parietal *x*; uninvolved [right] > involved [left]: frontal *x*, frontal *z*, and parietal *x*); thus, non-parametric Mann–Whitney *U*-tests were conducted for these dependent variables by group (ACLR and control). The other six dependent variables (involved [left] > uninvolved [right]: frontal *x*, parietal *y*, parietal *z*; uninvolved [right] > involved [left]: frontal *y*, parietal *y*, and parietal *z*) did not violate the assumptions, and independent *t*-tests were conducted for these dependent variables by group (ACLR and control). The same procedures for corrected *p*-values using the Benjamini–Hochberg approach were used in this analysis as previously described.

Peak voxel coordinate shifts for the involved [left] > uninvolved [right] limb contrast were further explored via non-parametric Kendall's tau-*b* correlations between the non-overlapping frontal and parietal regions [*x, y, z*] and involved limb isometric strength, time from surgery, IKDC, and KOOS Pain scores.

## 3 Results

### 3.1 Participant demographics

A total of 36 participants (18 ACLR and 18 controls) were enrolled in the study. A full list of participant demographics can be found in Table 1. Strength data for ACLR (*n* = 16) and control groups (*n* = 15) were based on a subset of the group totals, as not every participant was available for the strength assessment. There were no significant group differences for demographic variables of age, height, weight, activity level, and strength metrics (MVC). The ACLR group reported significantly lower scores for the IKDC and KOOS Pain relative to the control group, with a median difference of 12.64 (*p* < 0.001) and 8.33 (*p* < 0.001), respectively.

**Table 1:** Participant demographics.

	**ACLR**	**Control**	***P*-value**
Sex, (M:F)	8:10	7:11	–
Age, year^a^	21.00 (5.25)	22.00 (4.00)	0.18
Height, cm	171.72 ± 10.28	173.28 ± 10.87	0.66
Weight, kg	70.48 ± 15.70	69.66 ± 15.09	0.87
Tegner activity level scale^a^	7 (1.25)	7 (3)	0.20
Limb dominance (R:L)	16:2	16:2	–
Time from surgery, months	45.94 ± 7.55	–	–
Involved limb MVC, Nm/kg	2.03 ± 0.61	2.31 ± 0.75	0.26
Uninvolved limb MVC, Nm/kg	2.41 ± 0.73	2.30 ± 0.37	0.60
IKDC^a^	87.36 (15.23)	100.00 (2.30)	< 0.001
KOOS Pain^a^	91.67 (9.72)	100 (2.78)	< 0.001

MVC, max voluntary contraction. Height, weight, time from surgery, involved limb MVC, and uninvolved limb MVC were reported as mean ± standard deviation. Age, Tegner activity score, IKDC, and KOOS Pain were reported as median (interquartile range). The involved and uninvolved limbs of the ACLR group were compared to the left and right limbs, respectively, for controls.

^a^Non-parametric test (Mann–Whitney U-test).

### 3.2 Second-level analysis

#### 3.2.1 Involved [left] > uninvolved [right]

**Figure 1:**
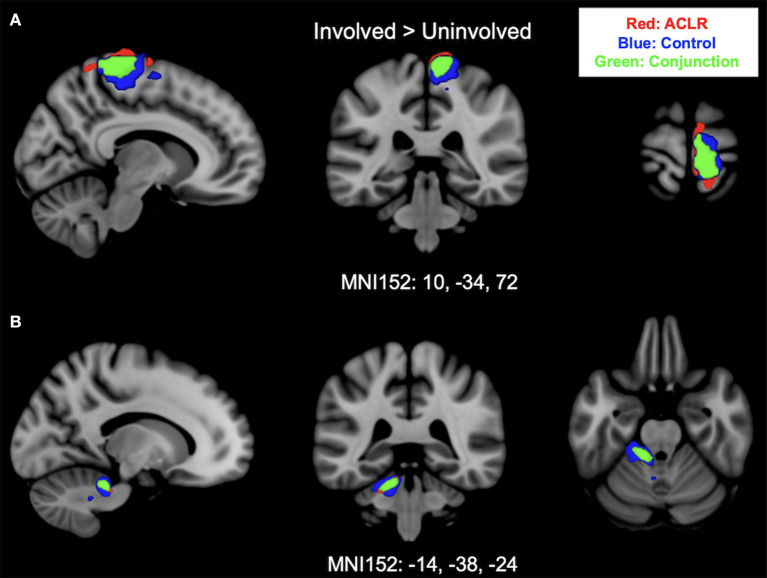
**(A)** Shows sensorimotor strip brain activity from the involved [left] > uninvolved [right] contrast. **(B)** Shows cerebellar brain activity from involved [left] > uninvolved [right] contrast. Results from the conjunction analysis are shown in green (areas of overlap between groups). Red represents unique brain regions activated by the ACLR group. Blue represents unique brain regions activated by the control group.

Brain activation for the involved [left] > uninvolved [right] contrast in the ACLR group produced two statistically significant clusters (Figure 1). Cluster 1 was in the right sensorimotor strip (voxels: 952, *z*_max_ = 5.88, MNI_xyz_: 8, −30, 78, *p* < 0.001), and cluster 2 was in the left cerebellum (voxels: 238, *z*_max_ = 4.9, MNI_xyz_: −14, −38, −24, *p* < 0.001). The control group produced two statistically significant clusters (Figure 1). Cluster 2 was in the left cerebellum (voxels: 404, *z*_max_ = 5.39, MNI_xyz_: −22, −34, −30, *p* < 0.001), and cluster 1 was in the right sensorimotor strip (voxels: 1,222, *z*_max_ = 5.59, MNI_xyz_: 10, −30, 58, *p* < 0.001).

#### 3.2.2 Uninvolved [right] > involved [left]

**Figure 2:**
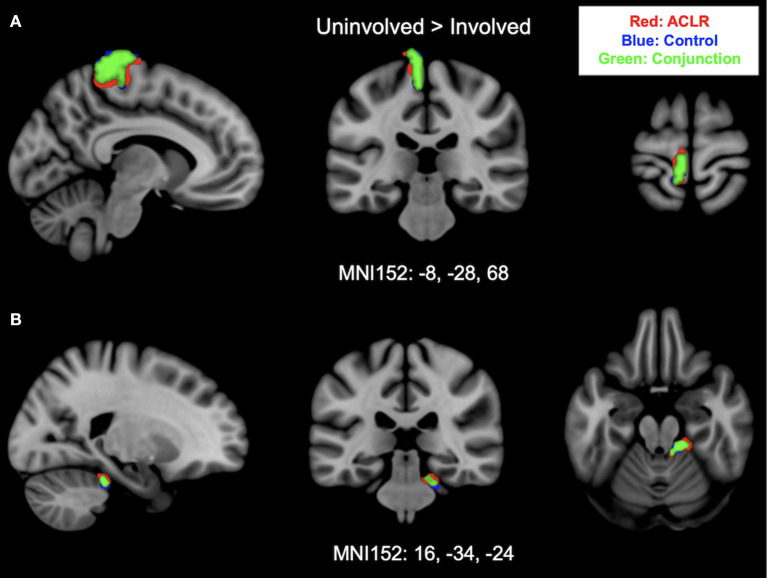
**(A)** Shows sensorimotor strip brain activity from uninvolved [right] > involved [left] contrast. **(B)** Shows cerebellar activity and brain activity from uninvolved [right] > involved [left] contrast. Results from the conjunction analysis are shown in green (areas of overlap between groups). Red represents unique brain regions activated by the ACLR group. Blue represents unique brain regions activated by the control group.

Brain activation for the uninvolved [right] > involved [left] contrast for the ACLR group produced two statistically significant clusters (Figure 2). Cluster 1 was in the left sensorimotor strip (voxels: 576, *z*_max_ = 5.09, MNI_xyz_: −10, −18, 74, *p* < 0.001), and cluster 2 was in the right cerebellum (voxels: 155, *z*_max_ = 4.5, MNI_xyz_: 26, −34, −24, *p* = 0.0102). The control group produced two statistically significant clusters (Figure 2). Cluster 1 was in the left sensorimotor strip (voxels: 428, *z*_max_ = 5.66, MNI_xyz_: −4, −34, 82, *p* < 0.001), and cluster 2 was in the right cerebellum (voxels: 296, *z*_max_ = 4.11, MNI_xyz_: 14, −36, −20, *p* < 0.001).

#### 3.2.3 Mean percent signal change

The primary finding for this analysis was that the ACLR group had greater activity in the sensorimotor strip relative to the control group for the involved [left] > uninvolved [right] contrast. For the involved [left] > uninvolved [right] contrast, the ACLR group had a statistically significant higher mean percent signal change (1.63 ± 0.55) in the sensorimotor strip compared with the control group [1.19 ± 0.55; *t*_(34)_ = 2.399, *p* = 0.044; Hedges' *g* = 0.78]. There were no statistically significant differences in either group for the cerebellum (involved [left] > uninvolved [right]). Additionally, for the uninvolved [right] > involved [left] contrast, there were no statistically significant differences in either group for either cluster (sensorimotor strip or cerebellum).

### 3.3 Conjunction analysis: overlapping areas

#### 3.3.1 Involved [left] > uninvolved [right]

Brain activation for the involved [left] > uninvolved [right] contrast between groups statistically overlapped in two clusters (Figure 1). Cluster 1 (Figure 1A) covered the right sensorimotor strip and included areas in the precentral gyrus and postcentral gyrus (voxels: 571, *z*_max_ = 4.74, MNI_xyz_: 10, −34, 72, *p* < 0.001). Cluster 2 (Figure 1B) included areas in the left cerebellum I–IV, cerebellum V, the left middle cerebellar peduncle, and the superior cerebellar peduncle (voxels: 170, *z*_max_ = 4.5, MNI_xyz_: −14, −38, −24, *p* = 0.006).

#### 3.3.2 Uninvolved [right] > involved [left]

Brain activation during uninvolved [right] > involved [left] contrast between groups statistically overlapped in two clusters (Figure 2). Cluster 1 (Figure 2A) covered the left sensorimotor strip and included areas in the precentral gyrus and postcentral gyrus: (voxels: 342, z_max_ = 4.31, MNI_xyz_: −8, −28, 80, *p* < 0.001). Cluster 2 (Figure 2B) included areas in the right cerebellum I–IV, cerebellum V, and the left middle cerebellar peduncle: (voxels: 118, *z*_max_ = 4.03, MNI_xyz_: 16, −34, −24, *p* = 0.037).

### 3.4 Conjunction analysis: sensorimotor strip non-overlapping areas

#### 3.4.1 Involved [left] > uninvolved [right]

As previously stated, the non-overlapping areas of the sensorimotor strip were split into frontal and parietal regions for both groups. The ACLR non-overlapping frontal areas included parts of the precentral gyrus, postcentral gyrus, and supplementary motor area (SMA), and the non-overlapping parietal areas included parts of the postcentral gyrus, superior parietal lobule, superior division of the lateral occipital cortex, and precuneus cortex (Table 2). For the control group, non-overlapping frontal areas included parts of the precentral gyrus and SMA, and the non-overlapping parietal areas included parts of the precentral gyrus, postcentral gyrus, and superior parietal lobule (Table 2). The ACLR group seemed to have more unique activation of superior parietal regions for the involved [left] > uninvolved [right] contrast relative to the control group.

**Table 2:** Regions of non-overlapping sensorimotor brain activity of the involved [left] > uninvolved [right] contrast.

	**Cluster**	**Brain regions (right)**	**Voxels**	**MNI 152 Peak voxel**			***Z*-max**
				***x***	***y***	***z***	
ACLR	Frontal	Precentral gyrus Postcentral gyrus^*^ Supplementary motor area Cerebral white matter Cerebral cortex Corticospinal tract	210	8	−30	78	5.88
	Parietal	Postcentral gyrus Superior parietal lobule Lateral occipital cortex (superior division)^*^ Precuneus cortex^*^ Cerebral white matter Cerebral cortex	171	12	−50	70	4.45
Control	Frontal	Precentral gyrus Supplementary motor area Cerebral white matter Cerebral cortex Corticospinal tract	307	20	−14	76	5.30
	Parietal	Precentral gyrus^**^ Postcentral gyrus Superior parietal lobule Cerebral white matter Cerebral cortex Corticospinal tract^**^	344	10	−30	58	5.59

^*^Denotes regions unique to the ACLR group.

^**^Denotes regions unique to the control group.

#### 3.4.2 Uninvolved [right] > involved [left]

The ACLR non-overlapping frontal area included parts of the precentral gyrus, and the non-overlapping parietal areas included parts of the precentral gyrus, postcentral gyrus, and precuneus cortex (Table 3). The non-overlapping frontal area of the control group included parts of the precentral gyrus and postcentral gyrus, and the non-overlapping parietal area included parts of the precentral gyrus and postcentral gyrus (Table 3).

**Table 3:** Regions of non-overlapping sensorimotor brain activity of the uninvolved [right] > involved [left] contrast.

	**Cluster**	**Brain regions (left)**	**Voxels**	**MNI 152 Peak voxel**			***Z*-max**
				***x***	***y***	***z***	
ACLR	Frontal	Precentral gyrus Cerebral white matter Cerebral cortex Corticospinal tract^*^	121	−10	−18	74	5.09
	Parietal	Precentral gyrus Postcentral gyrus Precuneus cortex^*^ Cerebral white matter Cerebral cortex Corticospinal tract	112	−10	−32	68	3.99
Control	Frontal	Precentral gyrus Postcentral gyrus Cerebral cortex	37	−4	−34	82	5.09
	Parietal	Precentral gyrus Postcentral gyrus Cerebral white matter Cerebral cortex Corticospinal tract	49	−8	−36	80	3.83

^*^Denotes regions unique to the ACLR group.

### 3.5 Conjunction analysis: cerebellum non-overlapping areas

#### 3.5.1 Involved [left] > uninvolved [right]

Overall, the ACLR group seemed to have a less diffuse pattern of cerebellar activity relative to the control group (i.e., fewer voxels and regions activated). For the ACLR group, non-overlapping cerebellum regions included parts of left lobules I–IV, left V, the left middle cerebellar peduncle, and the left superior cerebellar peduncle (voxels: 68, MNI_xyz_ = −22, −42, −30). The control group's unique areas of cerebellum activity included left lobules I–IV, left V, left VI, left IX, vermis IX, the left middle cerebellar peduncle, the left inferior cerebellar peduncle, and the left superior cerebellar peduncle (voxels: 234, MNI_xyz_ = −16, −36, −30).

#### 3.5.2 Uninvolved [right] > involved [left]

For the ACLR group, unique regions of cerebellar activity included parts of the right lobules I–IV, V, and the right middle cerebellar peduncle (voxels: 37, MNI_xyz_ = 26, −34, −24). The control group had unique cerebellar activity within parts of the right lobules I–IV, V, the right middle cerebellar peduncle, and the right superior cerebellar peduncle (voxels: 178, MNI_xyz_ = 10, −36, −20).

### 3.6 Peak voxel location of non-overlapping areas

Parametric data are presented as mean ± standard deviation, and non-parametric data are presented as median and IQR. Descriptive statistics of both second-level contrasts (involved [left] > uninvolved [right]) and (uninvolved [right] > involved [left]) for the frontal and parietal non-overlapping peak voxel coordinates can be found in Tables 4, 5.

**Table 4:** Descriptive statistics of peak voxel coordinates for non-overlapping regions (involved [left] > uninvolved [right]).

	**MNI 152**		
	***x***	***y***	***z***
**Frontal**			
ACLR	7.29 ± 3.55^*a^	−24.80 (7.70)^b^	77.20 (8.95)^*b^
Control	14.46 ± 4.81	−23.30 (10.68)	68.75 (6.95)
**Parietal**			
ACLR	10.50 (8.55)^b^	−40.74 ± 6.71^*a^	72.84 ± 4.85^*a^
Control	6.25 (8.60)	−34.89 ± 4.50	63.94 ± 5.25

^*^Significant difference between groups at the individual coordinate (p < 0.05).

^a^Mean ± standard deviation.

^b^Median (interquartile range).

**Table 5:** Descriptive statistics of peak voxel coordinates for non-overlapping regions (uninvolved [right] > involved [left]).

	**MNI 152**		
	***x***	***y***	***z***
**Frontal**			
ACLR	−9.00 (4.68)^b^	−24.56 ± 4.99^*a^	71.95 (6.72)^*b^
Control	−5.10 (6.08)	−30.80 ± 5.62	79.65 (2.50)
**Parietal**			
ACLR	−4.25 (3.98)^*b^	−36.03 ± 2.73^a^	60.99 ± 2.91^*a^
Control	−11.50 (6.88)	−37.31 ± 3.44	73.07 ± 3.44

^*^Significant difference between groups at the individual coordinate (p < 0.05).

^a^Mean ± standard deviation.

^b^Median (interquartile range).

#### 3.6.1 Frontal non-overlapping area (involved [left] > uninvolved [right])

The *x* coordinate was 7.17 mm more medial in the ACLR group compared to the control group [*t*_(34)_ = −5.09, *p* = 0.002 Hedges' *g* = 1.66]. There was no statistically significant difference in the *y* coordinate. The *z* coordinate was 8.45 mm more superior in the ACLR group compared to the control group (*U* = 55.00, *p* < 0.05, non-parametric ES = 0.56).

#### 3.6.2 Parietal non-overlapping area (involved [left] > uninvolved [right])

There was no statistically significant difference in the ACLR group compared to the control group for the *x* coordinate. The *y* coordinate was 6.24 mm more posterior in the ACLR group compared to the control group [*t*_(34)_ = −3.07, *p* < 0.05; Hedges' *g* = 1.0]. The *z* coordinate was 8.9 mm more superior in the ACLR group compared to the control group [*t*_(34)_ = 5.29, *p* < 0.05; Hedges' *g* = 1.72].

#### 3.6.3 Frontal non-overlapping area (uninvolved [right] > involved [left])

There was no statistically significant difference in the ACLR group compared with the control group for the *x* coordinate. The *y* coordinate was 6.24 mm more anterior in the ACLR group compared to the control group [*t*_(34)_ = 3.52, *p* < 0.05; Hedges' *g* = 1.15]. The *z* coordinate was 7.7 mm more inferior in the ACLR group compared to the control group (*U* = 303.00, *p* < 0.05, non-parametric ES = 0.74).

#### 3.6.4 Parietal non-overlapping area (uninvolved [right] > involved [left])

The *x* coordinate was 7.25 mm more medial in the ACLR group compared with the control group (*U* = 44.00, *p* < 0.05, non-parametric ES = 0.62). There were no statistically significant differences in the ACLR group compared with the control group for the *y* coordinate. The *z* coordinate was 12.08 mm more inferior in the ACLR group compared to the control group [*t*_(34)_ = −6.06, *p* < 0.05; Hedges' *g* = 3.71].

#### 3.6.5 Correlation of non-overlapping peak voxel location (involved [left] > uninvolved [right]) and involved limb behavioral measures

The shifts in peak voxel coordinates for the non-overlapping frontal and parietal regions (involved [left] > uninvolved [right]) were not significantly correlated with any of the behavioral measures of involved limb strength, time from surgery, IKDC, or KOOS Pain scores (Table 6).

**Table 6:** Correlations of non-overlapping peak voxel coordinates (involved [left] > uninvolved [right]) and behavioral measures.

	**Involved limb strength**		**Time from surgery**		**IKDC**		**KOOS Pain**	
	***r***	***p***	***r***	***p***	***r***	***p***	***r***	***p***
**Frontal**								
*x*	−0.067	0.719	0.210	0.246	0.013	0.939	−0.271	0.132
*y*	−0.067	0.718	0.042	0.816	0.080	0.648	0.189	0.297
*z*	−0.059	0.752	0.326	0.074	−0.094	0.593	0.007	0.969
**Parietal**								
*x*	0.133	0.471	0.084	0.642	−0.066	0.704	0.202	0.263
*y*	0.167	0.368	0.168	0.353	−0.159	0.361	0.007	0.969
*z*	0.100	0.589	0.154	0.395	−0.186	0.287	0.077	0.671

Data are presented as non-parametric Kendall's tau-b correlations. Involved limb strength data have been normalized to body weight.

## 4 Discussion

The purpose of the current study was to examine overlapping and non-overlapping brain activity for involved and uninvolved knee motor control between individuals with ACLR and non-injured controls. By examining functional activity overlap and non-overlap, as well as shifts in peak voxel location, we aimed to uncover novel insights into the neural adaptations following ACLR. A summary of all primary findings can be found in Table 7. Throughout the discussion, reference to the involved limb refers to the contrast of involved relative to uninvolved and not isolated involved limb movements, and reference to the uninvolved limb refers to the contrast of uninvolved relative to involved limb movements.

**Table 7:** Primary findings by variable and contrast.

**Variable**	**Contrast**	**Key findings**
Mean percent signal change	Involved > uninvolved	• ACLR group displayed statistically significant higher mean percent signal change in the sensorimotor cortex relative to the control group • No difference in the cerebellum
	Uninvolved > involved	• No difference in the sensorimotor cortex • No difference in the cerebellum
Areas of overlapping activation	Involved > uninvolved	• Statistically overlapped in two clusters ° Right sensorimotor area (precentral and postcentral gyrus) ° Left cerebellum (I–IV, V, middle cerebellar peduncle, and superior cerebellar peduncle)
	Uninvolved > involved	• Statistically overlapped in two clusters °Left sensorimotor area (precentral and postcentral gyrus)° Right cerebellum (I–IV, V, and middle cerebellar peduncle)
Unique areas of non-overlapping activation	Involved > uninvolved	• Frontal °ACLR: postcentral gyrus ° Control: no unique areas • Parietal ° ACLR: lateral occipital cortex (superior division) and precuneus cortex ° Control: no unique areas
	Uninvolved > involved	• Frontal ° ACLR: left corticospinal tract ° Control: no unique areas • Parietal ° ACLR: precuneus cortex ° Control: no unique areas
Peak voxel location of non-overlapping areas	Involved > uninvolved	• Frontal °ACLR group statistically more medial and superior • Parietal °sACLR group statistically more posterior and superior
	Uninvolved > involved	• Frontal ° ACLR group statistically more anterior and inferior • Parietal °ACLR group statistically more medial and inferior

Involved limb = left; uninvolved limb = right.

### 4.1 Sensorimotor cortex group differences

The conjunction analysis between ACLR and control groups provided novel data regarding statistically shared brain regions of activation during a knee motor control task. Shared anatomic regions for involved and uninvolved limb movement included the precentral and postcentral gyrus, which are the typical anatomical locations of the primary motor cortex (M1) and primary somatosensory cortex (S1), respectively. M1 is responsible for voluntary control of movement, while S1 receives primary sensory input and plays a role in proprioception (Sanes and Donoghue, 2000; Delhaye et al., 2018). The overall findings of the conjunction analysis are expected, as both M1 and S1 are necessary to generate coordinated limb movement for the knee motor control task. Regardless of overlapping sensorimotor strip activation, the ACLR group had a higher involved limb mean percent signal change relative to the uninvolved limb and controls. This finding aligns with previous fMRI studies involving individuals with ACLD and ACLR relative to healthy controls (Kapreli et al., 2009; Grooms et al., 2017). Similarly, in a transcranial magnetic stimulation (TMS) study, individuals with ACLR required stronger stimulation of the primary motor cortex to generate a motor-evoked potential, supporting impaired sensorimotor excitability in this group (Lepley et al., 2015). Thus, within the context of the motor control task, the higher mean percent signal change may indicate the ACLR group requires greater neural resources to produce the same knee flexion/extension movement relative to controls.

Overall, the ACLR group activated unique superior parietal regions during the motor control task, which may also indicate an involved limb neural compensation strategy. More specifically, the ACLR group displayed unique involvement in limb activation in the precuneus relative to the uninvolved limb and controls (Table 2). The precuneus has previously been identified as a multi-sensory region responsible for the direction of spatial attention during the execution of goal-directed movements (Wenderoth et al., 2005). Elevated precuneus activity has also been found for involved knee movement after ACLR, potentially contributing to the maintenance of function via the engagement of visual cognition (Chaput et al., 2022). The ACLR group also had unique involvement in limb activation in the superior division of the lateral occipital cortex (LOC). Although the LOC has known roles in object recognition (Grill-Spector et al., 2001), our participants had no direct visualization of their lower body during the motor control task, and thus, activation due to direct object recognition of the knee is unlikely in our task's context. However, the LOC and superior parietal regions have also been implicated in internal visualization of movement, and activation of these areas may be a result of imagining knee movement during the motor control task (Agnew et al., 2012; Pilgramm et al., 2016). In addition, Criss et al. (2020) found the LOC displayed increased activity during a hip–knee motor control task in an ACLR group relative to controls as well as increased functional connectivity with frontal and sensorimotor regions.

The precuneus and LOC both connect with the frontoparietal network, which is brought online during tasks involving attention to stimuli (Karten et al., 2013). One could argue that our motor control task requires attention to flex and extend the knee in time with the auditory metronome to stay on beat. Therefore, LOC and precuneus activity may be a neural compensation strategy requiring elevated attention to maintain involved knee motor control following ACLR. Interestingly, uninvolved limb movement also triggered unique activation in the precuneus, possibly indicating a neuroplastic change that is not isolated to the involved limb. These results corroborate a prior study of quadriceps gamma motor neuron dysfunction that implicates higher-level sensory integration demands (i.e., precuneus activity) for both the involved and uninvolved sides after ACLR contributing to functional loss (Konishi, 2011).

In further support of a unique neural strategy to control knee movement, both the involved and uninvolved limbs in the ACLR group showed frontal and parietal differences in peak voxel location compared to controls (Tables 4, 5). Shifts in peak voxel location or cortical organization are typical of individuals with chronic or persistent pain-altering joint afference (Tsao et al., 2008, 2011; Shanahan et al., 2015; Te et al., 2017). Previous research in LBP populations has shown a posterior and lateral shift in low back muscle representation relative to healthy controls (Tsao et al., 2008, 2011). These findings are partially congruent with the findings of the parietal non-overlap peak activation of the involved limb, in which the ACLR group had a more posterior and superior peak voxel location. The partial agreement between prior chronic LBP studies and the current investigation may be a result of the combined acute and chronic elements of ACL injury. Whereas the LBP studies point toward active pain and disrupted afference as a primary driver of neuroplasticity (Tsao et al., 2008, 2011), our ACLR group's brain activity was not influenced by pain (see Supplementary material). Thus, while possible pain memory or history might be influencing the results, loss of joint afference likely contributes to a greater degree than pain.

Shifts in peak voxel location of non-overlapping areas seen in our data may suggest a loss of discrete organization of the ACLR sensorimotor strip due to afferent loss from the native ACL. Prior research has identified deficits for involved limb proprioception tasks 6–24 months following ACLR (Fleming et al., 2022). However, our ACLR cohort was on average 5 years post-surgery, and evidence has also shown no differences in involved limb proprioception task performance when compared to healthy controls (Nakamae et al., 2017). Alternatively, rehabilitation therapies and prolonged time from surgery may result in behavioral changes, such as compensatory postural control strategies that shift cortical representation following ACLR (Gokeler et al., 2010; Alejandra Díaz et al., 2022).

Significant shifts in the involved limb *z* coordinate were also observed (8.45 mm superior shift within the sensorimotor cortex for the ACLR group) relative to the uninvolved limb. In a previous study, the involved limb corticospinal tract of those with ACLR was found to have both lower volume and excitability relative to the uninvolved side (Lepley et al., 2020). Therefore, we propose that the ACLR group may shift cortical representation in the *z* plane to overcome microstructural changes in the involved limb corticospinal tract to maintain motor control.

Overall, the shifts for involved limb peak voxel location in the non-overlapping frontal and parietal areas were independent of strength, time from injury, and patient-reported measures of knee function (Table 6). This finding adds to our understanding of why achieving quadriceps strength limb symmetry does not normalize motor performance or coordination such as gait or landing, as the brain activity for knee joint movement might still be asymmetric (Palmieri-Smith and Lepley, 2015; Schmitt et al., 2015; Lisee et al., 2019, 2022; Arhos et al., 2021; Kotsifaki et al., 2022). Future research may wish to explore other factors related to shifts in peak voxel location for the ACLR limb, such as deafferentation or rehabilitation therapies.

### 4.2 Cerebellar group differences

The conjunction analysis also provided data regarding statistically shared cerebellar activity and unique areas of activation between ACLR and control groups. Shared anatomic regions of brain activity for the involved limb included the left cerebellum I–IV, cerebellum V, left middle cerebellar peduncle, and superior cerebellar peduncle. Similar regions were active during uninvolved limb movement, as both groups statistically overlapped in areas of the right cerebellum I–V, cerebellum V, and right middle cerebellar peduncle. These areas of cerebellar activity are typical for sensorimotor tasks (Stoodley and Schmahmann, 2010; Stoodley et al., 2012) and indicate an appropriate neural response to the knee motor control task.

In addition to shared brain activity between groups, unique cerebellar activation was also identified. For involved limb function, the ACLR group had a more focal anatomical region of cerebellar activity. The control group had a more diffuse activation of cerebellar regions, including unique activation of the cerebellum left VI, left IX, vermis IX, vermis *x*, and the left inferior cerebellar peduncle. The vermal region is part of the spinocerebellum, a functional zone responsible for the motor coordination of gross limb movement (Unverdi and Alsayouri, 2023). Additionally, the inferior cerebellar peduncle is a primarily afferent white matter tract that relays proprioceptive information to the cerebellum to inform sensorimotor prediction and error correction (Jossinger et al., 2020). For uninvolved limb movement, the control group activated unique regions of the right superior cerebellar peduncle, an efferent white matter pathway that relays proprioceptive information from the cerebellum to the cortex (Pijnenburg et al., 2014). Overall, the ACLR group activated fewer unique sub-regions of the cerebellum relative to the control group, including the superior cerebellar peduncle. Previous research in individuals with a history of lateral ankle sprains found lower fractional anisotropy and higher radial diffusivity of the superior cerebellar peduncle (Terada et al., 2019), suggesting that joint injury could lead to alterations in cerebellar white matter microstructure. The results of this study, along with the unique activation of the superior cerebellar peduncle in the control group, suggest that microstructural changes in the cerebellar white matter may contribute to the less diffuse pattern of activation seen in the ACLR group.

The ACLR group exhibited a higher mean percent signal change in the sensorimotor cortex and less diffuse activation of unique cerebellar regions, further supporting a distinct limb coordination strategy compared to the control group. Previous electroencephalography (EEG) research in patients with ACLR has shown higher activation of frontal Theta frequencies during a force control task relative to control subjects (Baumeister et al., 2011), suggesting either more cognitive control of movement or a loss of cerebellar automaticity. Individuals after ACLR may rely more on cortical control than non-injured people who use a more cerebellar-regulated strategy to perform the same knee movements. Furthermore, other fMRI studies examining brain activation in ACLD and ACLR groups have found decreased activation in the ipsilateral (involved limb) cerebellum relative to healthy controls (Kapreli et al., 2009; Grooms et al., 2017). Although the current study found no differences in mean percent signal change, one possible reason for the lack of cerebellar findings in the current study may stem from the nature in which the second-level between-limb contrasts were carried out. Prior studies employed a directly involved knee vs. control contrast, whereas our study compared ACLR to controls after the respective within-group involved [left] > uninvolved [right] and uninvolved [right] > involved [left] limb contrasts. Thus, the nature of the analyses may have removed cerebellar activity. Additionally, as an *a priori* power analysis was not conducted, the lack of cerebellar findings may also be due to our sample being underpowered.

### 4.3 Limitations

There are several limitations to consider in this study. This study employed a cross-sectional design, so observed differences in neural activity or organization may not be solely attributable to ACLR but may have existed prior to injury or surgery. Future studies should consider a longitudinal design to better understand neural activity and organizational changes following ACL injury and ACLR. There is a possibility that the use of two different MRI scanners could introduce some variability in the data; however, the same parameters and techniques were used at both sites. Additionally, the data violated normality assumptions, but non-parametric analyses were used to address this issue. A larger sample size that is randomly drawn from the population might help to better address these concerns. Additionally, an *a priori* power analysis was not conducted for this study as it was a secondary data analysis from a larger study. Finally, one could argue that the nature of the knee motor control task is rather simplistic in design and may not translate to real-world scenarios or sport-specific movements. The task was designed as such to minimize participant head motion during fMRI scanning and preserve data integrity. Future studies may consider increasing the complexity of the lower extremity task while also preserving data quality.

## 5 Conclusion

To our knowledge, this study is the first attempt to provide insights into the organization of the sensorimotor cortex and cerebellum in individuals who have undergone ACLR for both the involved and uninvolved limbs. This study investigated overlapping and unique brain activation for involved and uninvolved knee motor control between ACLR and control groups. In the ACLR group, the involved limb had unique activation in areas responsible for sensory integration, potentially indicating a compensatory neural strategy to maintain motor control following the loss of afferent information from the native ACL, rehabilitation, or other behavioral change. Additionally, these compensatory strategies may not be isolated to the involved limb, as the uninvolved limb also activates unique brain regions relative to the control group. Future longitudinal research may wish to explore the exact mechanism that triggers reorganization in individuals with ACLR by gathering fMRI data prior to ACL injury and ACLR. Other methods to determine cortical reorganization might include models of knee deafferentation or examining the training effect following a bout of rehabilitation. Researchers may also try to identify specific structural changes in gray matter volume and white matter microstructure relative to healthy controls. Longitudinal research and prospective injury data are needed to confirm our results and explore the driving mechanism of cortical reorganization in individuals with ACLR.

## Data availability statement

The raw data supporting the conclusions of this article will be made available by the authors upon request, without undue reservation.

## Ethics statement

The studies involving humans were approved by the Institutional Review Boards at The Ohio State University and the University of Connecticut. The studies were conducted in accordance with the local legislation and institutional requirements. The participants provided their written informed consent to participate in this study.

## Author contributions

AS: Writing – original draft, Writing – review & editing, Conceptualization, Formal analysis, Methodology, Visualization, Data curation, Validation. HK: Conceptualization, Formal analysis, Methodology, Writing – review & editing, Visualization. AL: Data curation, Investigation, Writing – review & editing, Funding acquisition, Project administration, Resources, Supervision. JO: Supervision, Writing – review & editing, Data curation, Funding acquisition, Investigation, Project administration. CC: Conceptualization, Writing – review & editing. JS: Formal analysis, Writing – review & editing, Supervision. DG: Conceptualization, Data curation, Formal analysis, Funding acquisition, Investigation, Methodology, Supervision, Writing – review & editing, Project administration, Resources.
