# Supplementary material for: Organization of sensorimotor activity in anterior cruciate ligament reconstructed individuals: an fMRI conjunction analysis
Source: Front Hum Neurosci. 2023 Nov 24;17:1263292. doi: 10.3389/fnhum.2023.1263292 (PMC10704895; doi:10.3389/fnhum.2023.1263292)
Supplement: Supplementary file 1 [file Data_Sheet_1.docx]

Supplementary Material

# Supplementary Data

To explore the effect of KOOS pain scores on brain activity for involved [left] limb movement, we conducted a single group average of the left limb motor control task both with and without KOOS pain as a covariate (Supplemental Figures 1-2). Independent samples t-tests were conducted to determine if there was a statistically significant mean difference in the number of voxels activated between the two analyses (*p*<.05).

Covarying for KOOS pain scores did not significantly alter brain activity for involved [left] limb motor control in the ACLR (t_(16)_=.242, p=.81; Hedges’ g=.12) or control groups ((t_(3)_=.292, p=.79; Hedges’ g=.19). Because KOOS pain did not significantly alter brain activity, we chose to exclude it as a covariate in all analyses.

Individual item counts on the KOOS pain subscale for the ACLR group are located in Supplemental Table 1. Of note, none (0.0%) of our ACLR participants experienced severe or extreme pain with fully bending or straightening their involved limb, which was essential to the motor control task.

# Supplementary Figures and Tables

**Supplemental Figure 1.** Red represents the single ACLR group average of brain activity for the involved [left] motor control task. Blue represents the single ACLR group average of brain activity for the involved [left] motor control controlling for KOOS pain. Green represents the overlapping brain activity of analyses with and without the KOOS pain covariate.

**Supplemental Figure 2.** Red represents the single Control group average of brain activity for the involved [left] motor control task. Blue represents the single Control group average of brain activity for the involved [left] motor control controlling for KOOS pain. Green represents the overlapping brain activity of analyses with and without the KOOS pain covariate.

| **Supplemental Table 1.** ACLR Group Counts and Percentages of KOOS Pain Items | | | | | |
| --- | --- | --- | --- | --- | --- |
|  | **Never** | **Monthly** | **Weekly** | **Daily** | **Always** |
| P1. How often do you experience knee pain? | 3 (16.7%) | 8 (44.4%) | 6 (33.3%) | 1 (5.6%) | 0 (0.0%) |
| *What amount of knee pain have you experienced in the last week during the following activities?* | **None** | **Mild** | **Moderate** | **Severe** | **Extreme** |
| P2. Twisting/pivoting on your knee | 11 (61.1%) | 5 (27.8%) | 2 (11.1%) | 0 (0.0%) | 0 (0.0%) |
| P3. Straightening knee fully | 14 (77.8%) | 2 (11.1%) | 2 (11.1%) | 0 (0.0%) | 0 (0.0%) |
| P4. Bending knee fully | 12 (66.7%) | 6 (33.3%) | 0 (0.0%) | 0 (0.0%) | 0 (0.0%) |
| P5. Walking on a flat surface | 17 (94.4%) | 1 (5.6%) | 0 (0.0%) | 0 (0.0%) | 0 (0.0%) |
| P6. Going up or down stairs | 14 (77.8%) | 4 (22.2%) | 0 (0.0%) | 0 (0.0%) | 0 (0.0%) |
| P7. At night while in bed | 15 (83.3%) | 3 (16.7%) | 0 (0.0%) | 0 (0.0%) | 0 (0.0%) |
| P8. Sitting or lying | 17 (94.4%) | 1 (5.6%) | 0 (0.0%) | 0 (0.0%) | 0 (0.0%) |
| P9. Standing upright | 16 (88.9%) | 2 (11.1%) | 0 (0.0%) | 0 (0.0%) | 0 (0.0%) |
