# Supplementary figures and images for: Organization of sensorimotor activity in anterior cruciate ligament reconstructed individuals: an fMRI conjunction analysis
Source: Front Hum Neurosci. 2023 Nov 24;17:1263292. doi: 10.3389/fnhum.2023.1263292 (PMC10704895; doi:10.3389/fnhum.2023.1263292)

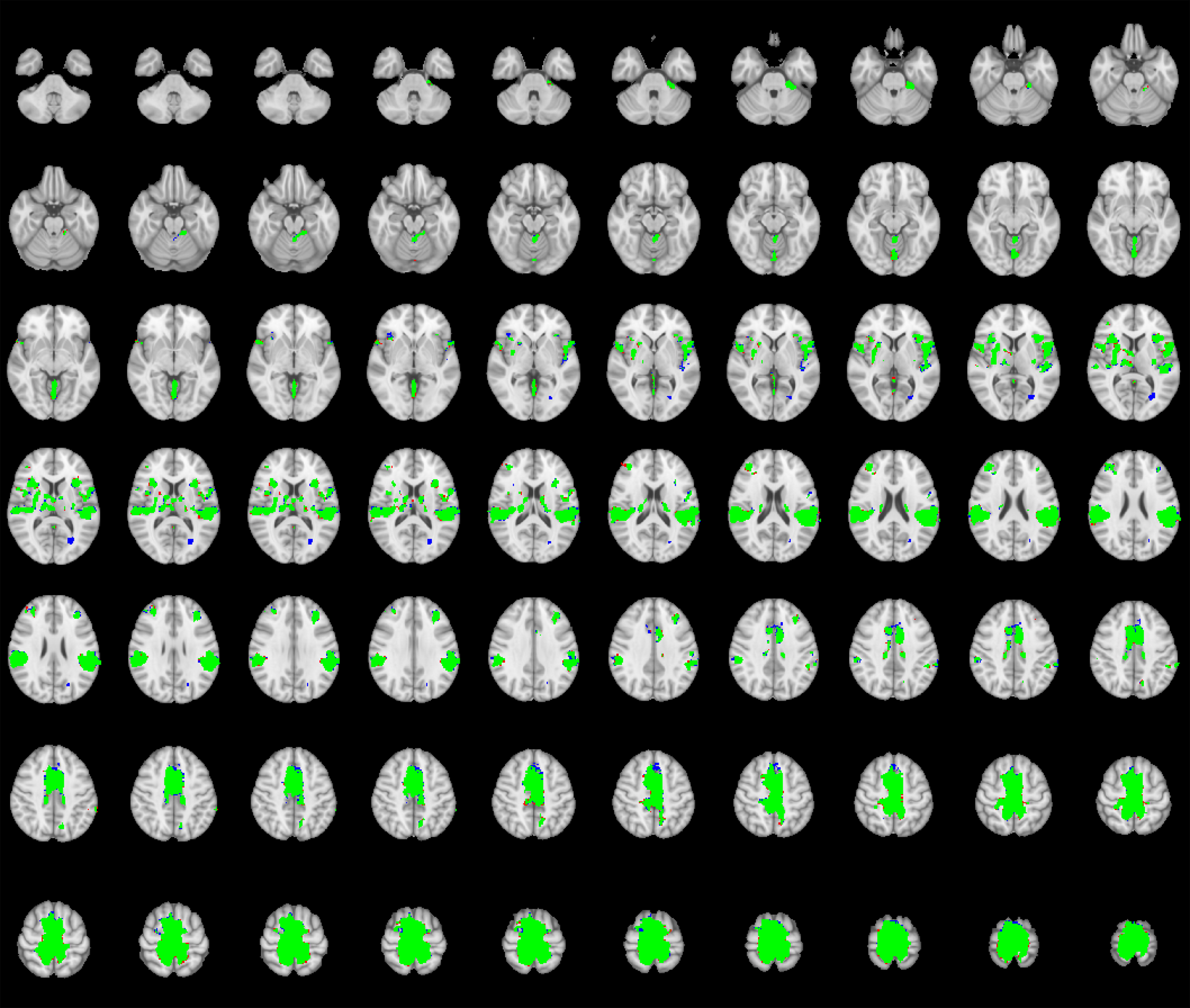

Supplement: Supplementary file 2 [file Image_1.TIFF]

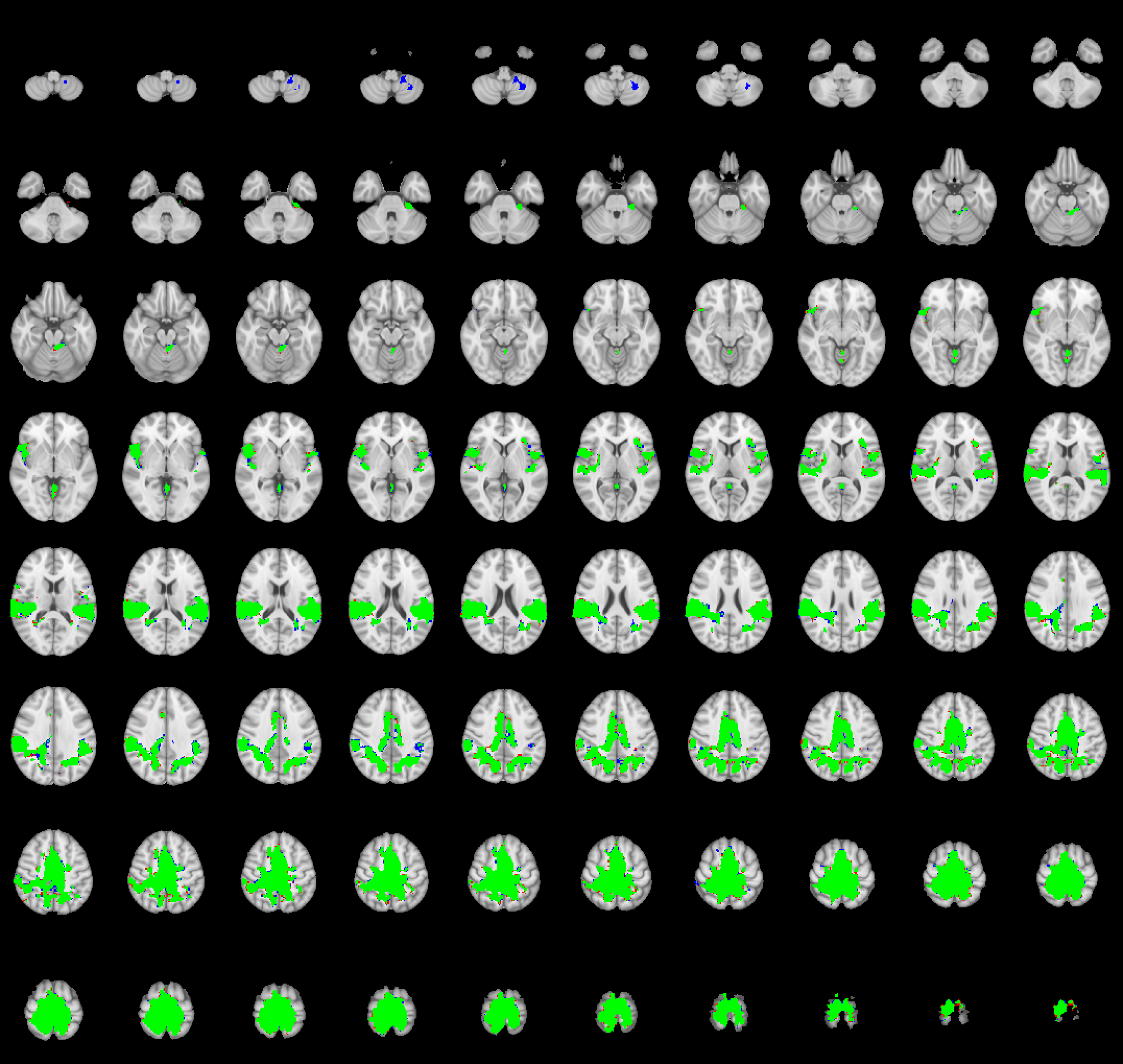

Supplement: Supplementary file 3 [file Image_2.TIFF]
